# Solid Dispersions of Gefitinib Prepared by Spray Drying with Improved Mucoadhesive and Drug Dissolution Properties

**DOI:** 10.1208/s12249-021-02187-4

**Published:** 2022-01-04

**Authors:** Wesam W. Mustafa, John Fletcher, Mouhamad Khoder, Raid G. Alany

**Affiliations:** 1 Drug Discovery, Delivery and Patient Care Theme, Department of Pharmacy, Kingston University London, Kingston upon Thames, KT1 2EE UK; 2 Department of Pharmacy, Al-Mustafa University College, Baghdad, Iraq; 3 School of Pharmacy, The University of Auckland, Auckland, New Zealand

**Keywords:** gefitinib, Eudragit S 100, PVP, HPMC, solid dispersion, colon-targeting

## Abstract

Gefitinib is a tyrosine kinase inhibitor that is intended for oral administration yet suffers poor bioavailability along with undesirable side effects. To enhance its solubility and allow colon targeting, gefitinib (ZD) and blends of different ratios of polymers (ternary dispersion) were prepared in organic solution, and solid dispersions were generated employing the spray drying (SD) technique. The methylmethacrylate polymer Eudragit S 100 was incorporated for colon targeting; polyvinylpyrrolidone (PVP) and hydroxypropyl methyl cellulose (HPMC) were utilised to improve the solubility of ZD. SEM, DSC, XRPD, FT-IR, dissolution and cytotoxicity studies were undertaken to characterise and evaluate the developed formulations. SEM images revealed that the rod-shaped crystals of ZD were transformed into collapsed spheres with smaller particle size in the spray-dried particles. DSC, FTIR and XRPD studies showed that ZD loaded in the spray-dried dispersions was amorphous. ZD dissolution and release studies revealed that while a significant (*P* < 0.05) increase in the ZD dissolution and release was observed from HPMC-based solid dispersion at pH 7.2 (up to 95% in 15 h), practically no drug was released at pH 1.2 and pH 6.5. Furthermore, the HPMC-based solid dispersions displayed enhanced mucoadhesive properties compared with PVP-based ones. Interestingly, cell viability studies using the neutral red assay showed that PVP and HPMC-based solid dispersions had no additional inhibitory effect on Caco-2 cell line compared to the pure drug.

## INTRODUCTION

Gefitinib (ZD) is a specific epidermal growth factor receptor (EGFR) tyrosine kinase inhibitor that inhibits signal pathways involved in the growth of solid tumour and metastasis. ZD was approved as a single agent for treatment of non-small cell lung cancer. The mechanism of action of ZD has been linked to its targeted inhibition to EGFR tyrosine kinase by binding to the adenosine triphosphate (ATP) (1). Hence, the role of the EGFR tyrosine kinase in stimulating the anti-apoptotic Ras signal transduction cascade is inhibited, and therefore, the tumour cell activities are also inhibited (2). For colorectal cancer treatment, studies on ZD have taken multiple paths. In patients with colon cancer, there is a theoretical rationale to the use of ZD. As in colorectal tumours where the EGFR is over-expressed, this over-expression is related with a worse prognosis (3).

Site-specific drug delivery has gained considerable interest over systemic therapy as it allows reducing drug dose and dose frequency by directing the drug at the disease spot and preventing metabolism of the drug, hence enhancing efficacy and reducing side effects (4, 5). Targeting the drug release to the colon for the local treatment of colonic disorders and cancers has appealed to researchers and clinicians (6, 7). Colon-targeted delivery has gained much popularity for treating local diseases of the colon such as ulcerative colitis and colon cancer. These targeted dosage forms can provide high local drug concentration with minimal systemic side effects. Colon cancer is one of the most commonly diagnosed cancers that causes cancer-related death worldwide (8, 9).

One of the most widely used approaches for developing successful colon-targeted drug delivery is coating drug particles with pH-sensitive polymers (such as Eudragit S100). Such a strategy would prevent premature and undesirable rupture of the polymeric membrane in upper parts of the gastrointestinal tract such as the stomach and intestine. Spray drying is a successful and widely used technique to prepare coated particulate drug delivery system for different purposes including colon-targeted drug delivery (10).

ZD is a weak base with pKa values of 5.4 and 7.2; its insoluble in water and can be considered as a Class II drug based on the Biopharmaceutical Classification Scheme. Accordingly, ZD has low solubility and high permeability (11–14). ZD is formulated as a tablet for oral delivery to treat lung cancer (15). The fact that ZD is a poorly water-soluble drug that is intended to treat colorectal cancer via oral delivery warrants the need for an oral formulation that is able to simultaneously achieve a dual purpose and enhance its solubility and deliver it specifically to the colon. Although colon cancer at stage 0 and stage 1 can be managed by surgical intervention, the oral administration of anticancer agent (i.e. ZD) is preferred by both health care professional and patient. Moreover, a successful colonic delivery of anticancer agent helps localise the drug at the target site and provide the colon area with a more soluble form of the drug in a controlled release profile.

It has been previously reported that the lack of free fluid in the transverse and descending colon contributes to the incomplete release and dissolution of the drugs leading to therapeutic ineffectiveness (16). Therefore, releasing the drug at the ileo-colonic segment is preferred where more fluids, hence the rationale for using a formulation with mucoadhesive characteristics. Furthermore, enhancing the drug dissolution rate is essential to allow complete drug release. The use of a solid dispersion, in which the drug is dispersed within one or more inert water-soluble carriers, is an established approach to overcome a poor dissolution profile (17). For ideal ileo-colonic targeting, the dosage form should effectively prevent drug release in the upper gastro-intestinal tract (GIT) only releasing the drug as it reaches the colon (18).

In this study, two hydrophilic polymers PVP and HPMC were selected as carriers for spray-dried solid dispersions to enhance the solubility and dissolution profile of ZD (19, 20). Furthermore, HPMC as a mucoadhesive polymer could potentially prolong contact time with the drug absorption surface to enhance tissue permeability of ZD and, thus, improve absorption and potentially increase efficacy and reduce adverse effects of the drug (21). Eudragit S 100, a pH-dependent polymer was selected as a secondary polymer in ternary dispersion to target the drug to the intended site (i.e. ileo-colonic site) (22). Eudragit S 100 is widely used in modified-release formulations (23).

Scanning electron microscopy, differential scanning calorimetry and X-ray diffractometry techniques were used to characterise the formulated solid dispersion and dissolution studies were performed over a range of biorelevant pH values to investigate the drug dissolution and release profiles. The *in vitro* model developed by Needleman *et al.*(24) was used to evaluate the mucoadhesive properties of selected formulations. Finally, neutral red assay was performed to assess the cytotoxicity of the pure ZD and ZD formulated in HPMC-bases solid dispersions on Caco-2 cell line.

## MATERIALS AND METHODS

### Materials

Gefitinib (ZD) (ZD1839) was purchased from Med Chem Express Co., Ltd. (Shanghai, China). The Caco-2 cell line, trifluoroacetic acid (TFA), foetal bovine serum (FBS), thiazolyl blue tetrazolium bromide, l-glutamine-penicillin-streptomycin solution, dimethyl sulfoxide (DMSO), Eagle’s minimum essential medium (EMEM), trypsin-EDTA solution, trypan blue solution (0.4%), neutral red and actinomycin D were all purchased from Sigma-Aldrich (Dorset, UK). Acetone, sodium hydroxide and absolute ethanol were obtained from VWR International (Leicestershire, UK). Hydrochloric acid, Dulbecco’s phosphate-buffered saline (PBS), sodium chloride, Dulbecco’s modified Eagle’s medium (DMEM), potassium dihydrogen orthophosphate, acetic acid glacial and Nunclon 96-well microplates were purchased from Fisher Scientific UK Ltd. (Loughborough, UK). Eudragit S 100 was provided by Evonik Röhm Gmbh (Essen, Germany). Kollidon K 30 (PVP) was kindly donated by BASF SE (Ludwigshafen, Germany). HPMC 603 (HPMC) was kindly given by Shin-Etsu Chemical Co., Ltd. (Tokyo, Japan).

### Solid Dispersions Preparation

Specific amount of ZD (1 g) was dissolved in a mixture of acetone (60 mL) and ethanol (40 mL). Then, PVP and/or HPMC (see Table 1 for quantities and compositions) were added gradually with stirring for 20 min to dissolve. Distilled water (20 mL) was then added to the PVP solution stirred solution. For HPMC mixtures of different polymer content, 10 mL of hot water and then 10 mL of cold water were added to the specified amount of HPMC. Finally, specified amounts of Eudragit S100 (see Table 1 for quantities and compositions) to yield a 1:9 final drug (10 g batch): polymer mass (w/w) ratios.

**Table 1:** Drug to Polymer Ratios of Formulations Investigated in This Study^*, **^

S. No	Formulation code	ZD (g)	Polymer content (g)		Eudragit S 100 (g)
			PVP	HPMC	
1	SDZDPS	1.0	7.2	-	1.8
2	SDZDHS	1.0	-	7.2	1.8
3	SDZDHS-M	1.0	-	7.1	1.9
4	SDZDHS-Z	1.0	-	7.0	2.0

^*^The formulated solid dispersions codes were constructed in a manner to better elucidate their constituents. For example, in ‘SDZDPS’ or ‘SDZDHS’, SD represents solid dispersions, while (ZDPS or ZDHS) represents solid dispersion constituents of gefitinib (ZD), PVP, Eudragit S 100 for ZDPS; and gefitinib (ZD), HPMC, Eudragit S 100 for ZDHS

^**^M or Z indicates the polymer content for HPMC and Eudragit S 100 in the dispersions (as shown in the above table)

The resulting mixture was stirred for a further 30 min before being spray-dried using mini spray dryer (Büchi B-290, Büchi Labortechnik AG, Switzerland). The spray drier was set off with the inlet temperature of 90°C, feeding rate of 3 mL/min, atomising air pressure of 3000 psi and rate of nitrogen gas of 600 L/h with 100% aspiration. The percentage yield was between 84 and 86%.

### Scanning Electron Microscopy

A Zeiss EVO 50 Scanning Electron Microscope (SEM) (Oberkochen, Germany) was employed to investigate the morphology and particle size of ZD, PVP, HPMC, Eudragit S100 and the solid dispersions. An SEM sample was prepared by coating the particles with a thin layer of gold to ensure adequate conductivity to the sample’s surface using a Polaron SC500 sputter coater (Polaron Equipment, Watford, UK) under argon. Powdered samples were mounted on adhesive carbon tape fixed on metal stubs and examined under low vacuum mode at an acceleration voltage of 20 keV.

### X-ray Diffractometry (XRD)

A BrukerAXS D8 X-ray diffractometer (Karlsruhe, Germany) was used to obtain the diffraction patterns of the raw materials (ZD, PVP, HPMC) and solid dispersions. The voltage and current used were 20 kV and 5 mA, respectively. The scan region was from 11° to 30° 2θ with a step size of 0.2° 2θ and a time per step 0.18 s at room temperature. The resultant diffraction patterns were analysed using DIFFRAC plus XRD commander software.

### Differential Scanning Calorimetry (DSC)

ZD, PVP, HPMC and the solid dispersions were thermally evaluated using a DSC 822e apparatus (Mettler-Toledo Ltd., Leicester, UK). Accurately weighed sample (3–10 mg) was loaded into a sealed pinhole aluminium crucible pan and placed on the top of the sample holder, under nitrogen flow of 10 mL min^−1^. All samples were heated from room temperature up to a maximum of 230°C at 20°C/min, and results were analysed using STAReSW 10.00 software.

### Fourier-Transform Infrared (FT-IR) Spectroscopy

Thermo Fisher Scientific FT-IR spectrometer (Nicolet iS5, iD5 advanced attenuated total reflectance (ATR), USA) was employed in this study. A collection of the spectrum occurred in the range of 4000 to 600 cm^−1^. A tiny amount of sample was located in the centre of the diamond part, and then a stainless-steel bar was located on the top of the sample surface and pushed using a screw. The collection of each spectrum included an average of 64 scans, and an average of three measurements was employed. The data were collected using OMNIC (Omnic version 8.2, USA) software.

### Dissolution Studies

Dissolution studies were performed at 37°C ± 0.5°C using a Type II dissolution apparatus (Caleva Ltd., Dorset, UK), with 900 mL of different buffered medium (phosphate buffer pH 6.5 and 7.2 and hydrochloric acid 0.1 N for pH~1.2) using the paddle method with a rotation speed of 100 ± 2 rpm (23). The experiment was conducted using a pump with an eight-channel head (Automated Lab Systems Ltd., Berkshire, UK), linked to a spectrophotometer (heλos αUV-vis, Waltham, MA, Thermo Scientific Inc.). A sample of 20 mg of ZD was placed manually in a hard gelatine capsule (size 0), and a sample equivalent to 20 mg ZD formulation (200 mg) was placed manually in a hard gelatine capsule (size 00) and then dropped using a capsule sinker into the dissolution medium. An aliquot (1 mL) of dissolution medium was transferred to the cuvette automatically at a programmed time interval to determine the absorbance. The percentages of released ZD were calculated by the linear regression equation obtained from the calibration curves prepared using the respective dissolution media.

### Solubility Studies

The solubility of the crystalline ZD was tested by dissolving an excessive amount of drug into each of the solvents (10 mL) (phosphate buffer pH 6.5 and 7.2 and hydrochloric acid 0.1 N for pH~1.2) in a small beaker. After 24 h of continuous stirring, the solution was left for 24 h for equilibration and then filtered through a 0.45 μm polypropylene syringe-driven microfilter. Ultraviolet-visible (UV-vis) spectrophotometer (Varian Cary Bio 100, Agilent Technologies, USA) was used to quantify the drug in the solutions.

### Mucoadhesion Study

A modified version of the *in vitro* mucoadhesion model reported by Needleman and Smales was employed. The duration of mucoadhesion of selected polymeric solid dispersion systems (SDZDPS, SDZDHS) on a mammalian excised intestinal tissue was measured (24). In brief, a small piece of sheep’s large intestine tissue (freshly obtained from a local butcher) was attached to an iron mesh fixed on a cylindrical base. This system was then placed into a plastic falcon tube. The tube was filled with different media (pH 1.2, 6.5, 7.2) up to the tissue (24, 25). The whole setup was transferred into a shaking water bath at 37°C for 10 min to equilibrate. A sample formulation (10 mg) was sprinkled on the tissue. The sample was equilibrated on the tissue at 37°C for half an hour. Afterwards, a glass cover slip was placed on top of the formulation and a constant pressure was applied with finger for 10 s. The tube containing the tissue and formulation with the cover slip was mounted horizontally in the shaking water bath that was set at 100 strokes per minute. For the first 30 min, the tube was observed every 120 s for any possible detachment of the cover slip and then every 20 min for up to 20 h. The average cove slip detachment time was determined for three separate measurements.

### Cell Viability Assay

Caco-2 cells were seeded in 96-well plates (20,000 cells/well) and incubated for 24 h. A specific volume (20 μL) of stock solutions (10 mM) of pure drug, solid dispersions with or without drug was added to 180 μL media to obtain a final ZD concentration of 1000 μM. Then, a 1-log dilutions were done to obtain the following concentration range (0.001 μM, 0.01 μM, 0.1 μM, 1.0 μM, 10 μM and 100 μM). The positive control used in this study was actinomycin D (100 μM). The Caco-2 cell lines were treated for 24 h, after which they were incubated for 3 h with 40 μg/mL neutral red in the medium. The cells were later washed with D-PBS and the dye was extracted using de-stain solution (1% acetic acid in 50% hydroalcoholic acid solution). The absorbance was measured at 540 nm using a BioTek plate reader (Epoch Microplate Spectrophotometer, Winooski, United States).

## RESULTS AND DISCUSSION

### Scanning Electron Microscopy

**Fig. 1:**
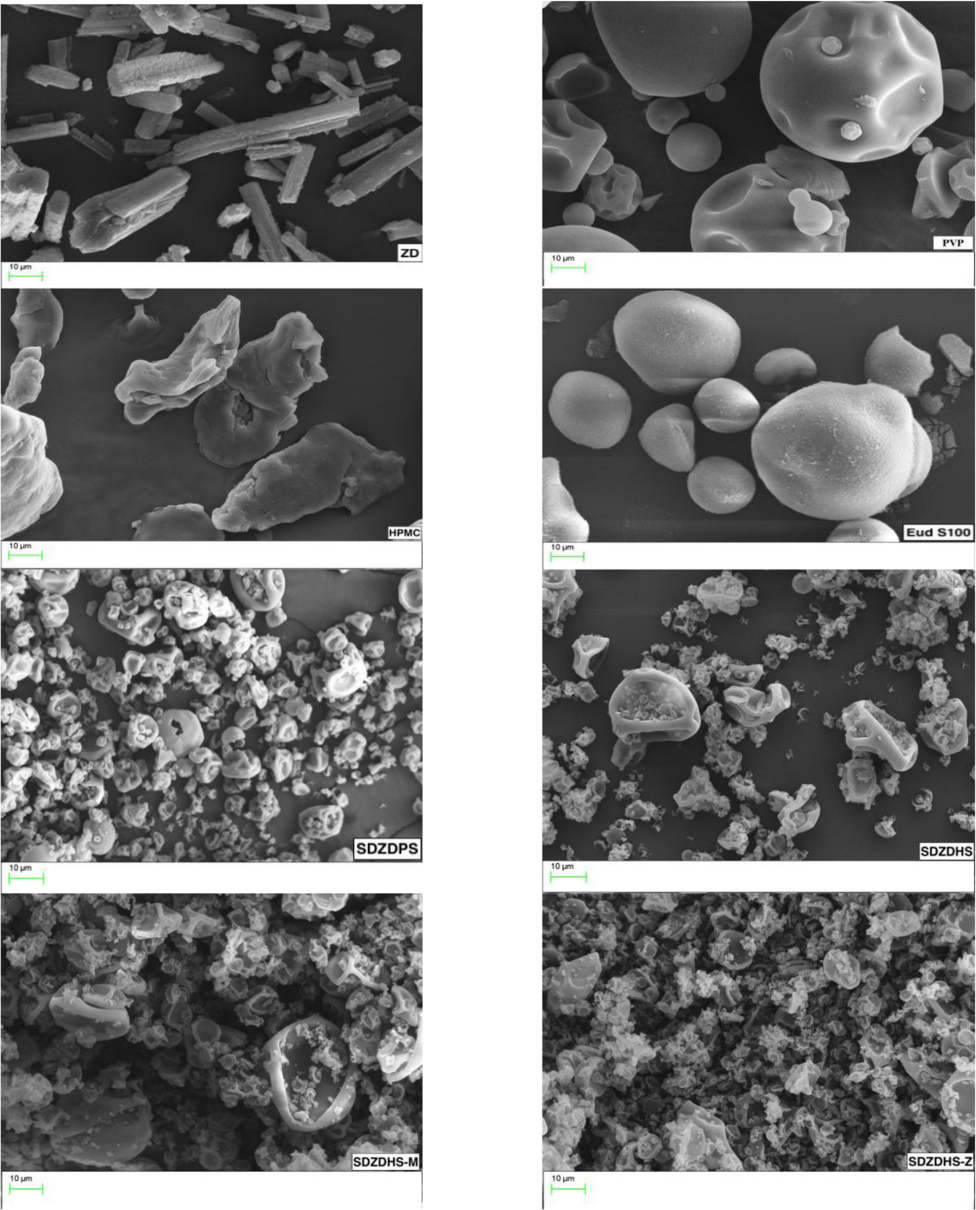
SEM images displaying the morphology of ZD, PVP, HPMC, Eud S100, SDZDPS, SDZDHS-M and SDZDHS-Z

SEM was employed to examine the surface morphology of ZD, PVP, HPMC and Eudragit S100 in their pure states, and solid dispersions prepared by the spray drier (Figure 1). While pure ZD SEM image reveals rod-shaped coarse (large) crystals, PVP appeared as irregular spherical particles, HPMC as irregular flake particles and Eud S100 as regular spherical particles.

The SDZDPS dispersion appeared as agglomerates of collapsed spheres, while SDZDHS, SDZDHS-M and SDZDHS-Z consisted of clusters of particles. All ternary solid dispersions had a markedly smaller particle size compared with untreated pure ZD. These favourable dimensions and morphology contribute to the larger surface areas in contact with the dissolution media *in vivo* and hence are likely to have a significant impact and increase dissolution rate of the drug from these spray-dried formulations (26).

### X-ray Diffractometry

**Fig. 2:**
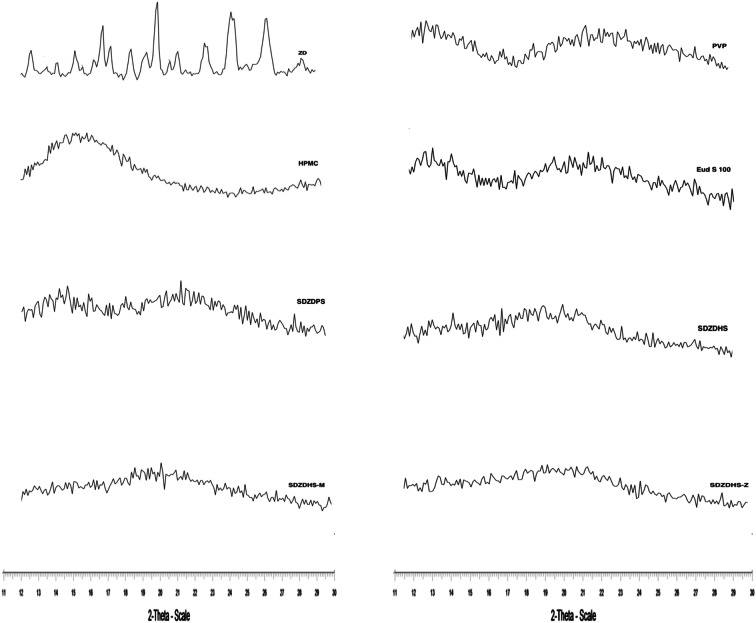
X-ray powder diffractograms of ZD, PVP, HPMC, Eudragit S 100 and the solid dispersions

Figure 2 shows the diffractograms of pure ZD, PVP, HPMC, Eudragit S100 and the spray-dried solid dispersions. Several diffraction peaks are clear in the diffractogram of ZD at 15.1°, 16.1°, 18.6°, 19.2°, 20.6°, 22.5°, 24.2° 26.2° and 26.4°. Similar peaks were reported in previous study which were attributed to drug crystallinity (27). PVP, HPMC and Eudragit S100 showed no sharp peaks in their diffractograms which is attributed to their availability in the amorphous form. None of the ZD peaks can be observed in any of the ternary solid dispersions. This observation indicates that ZD was successfully converted into an amorphous or molecular dispersed form (28). The amorphous form is likely to show higher saturation solubility than the crystalline forms due to its higher free energy levels (29). For example, amorphous state formation of nimesulide using PVP solid dispersion resulted in greater enhancement of dissolution rates and solubility of this insoluble drug (30).

### Differential Scanning Calorimetry (DSC)

**Fig 3:**
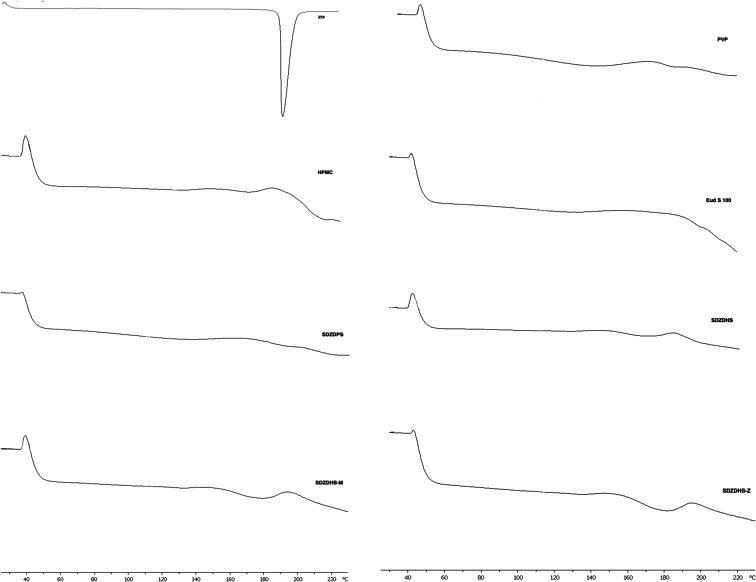
DSC thermograms of ZD, PVP, HPMC, Eud S 100 and their solid dispersion

DSC has been employed to investigate the solid-state changes of ZD in the formulated solid dispersions. Figure 3 displays the DSC thermograms of ZD, PVP, HPMC, Eudragit S100 and their solid dispersions. ZD shows a sharp endothermic melting peak at 192.01±0.06°C, which confirms its crystallinity (31). On the other hand, thermograms for PVP, HPMC and Eudragit S 100 show the lack of melting peak which confirm they are in amorphous forms. Figure 3 shows that typical sharp and strong ZD melting peak was completely missed and replaced with a very shallow and broad peak in all solid dispersions thermograms which suggests the successful drug conversion into its amorphous form. Analysis by the DSC using heat-cool-heat cycle provided the glass transition temperature (*Tg*) of each of the polymers and dispersions (Table 2). All ternary dispersions have shown a single glass transition (*Tg*) with the lack of an endothermic event. This indicates the presence of ZD in the amorphous state and formation of homogeneous dispersions of ZD within these polymers. These results agree with the XRD data, which indicate that ZD was amorphous in all of the solid dispersion formulations.

**Table 2:** ***Tg*** Values of PVP, HPMC, Eud S 100 and Solid Dispersions

Dispersion constituent	*Tg* (°C)
PVP	160.21 ± 0.57
HPMC	125.91 ± 0.31
Eudragit S 100	160.61 ± 0.54
SDZDPS	152.37 ± 0.25
SDZDHS	122.49 ± 1.34
SDZDHS-M	122.95 ± 0.93
SDZDHS-Z	122.17 ± 0.43

### Fourier-Transform Infrared Spectroscopy (FT-IR)

**Fig. 4:**
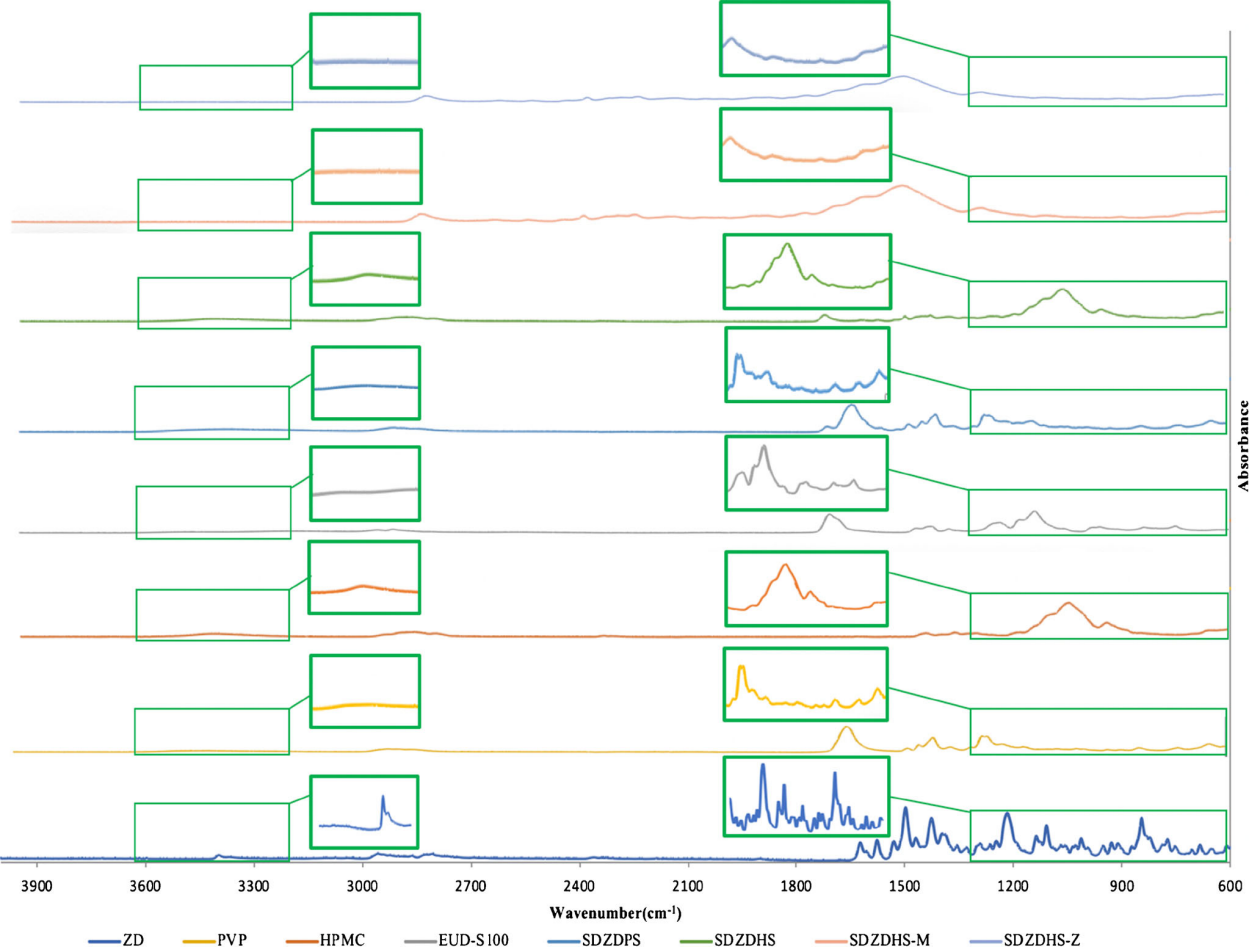
IR spectra of ZD, PVP, HPMC and EUD-S100, and their solid dispersion over the spectral region between 600 and 4000 cm^−1^

The IR spectrum of ZD (Figure 4) shows a sharp peak at 3397 cm^−1^ that was assigned to the secondary amine group (the secondary amine stretching range is 3500–3100 cm^−1^) (32). Polyvinylpyrrolidone (PVP) has an amide carbonyl group in its structure, which shows a characteristic sharp peak (Figure 4) at 1664 cm^−1^(33). HPMC has residual free OH groups, which produces a peak at 3455 cm^−1^(33). The carbonyl group of Eudragit S100 shows a peak (Figure 4) at 1724 cm^−1^(34). In probing interactions between the drug and polymers, the spectra of pure ZD were used in combination with spectrum of the polymers as references to compare with those of the solid dispersions. The spectra (Figure 4) of the solid dispersion show differences when compared with the pure drug (Figure 4). The ZD solid dispersion secondary amine peak disappeared and a broad peak at the same wavenumber range was observed in all dispersions, which is indicative of the interaction (hydrogen bonds) between the O- and NH- groups of the drug and polymers. Further, the disappearance of fingerprint region of IR spectra of ZD at 1300 to 600 cm^−1^ with solid dispersed mixtures could be ascribed to drug-excipient interactions, alteration of drug crystallinity and alteration of main functional groups. These favourable electrostatic interactions are likely to support the formation of amorphous/molecular dispersion and are in agreement with DSC and XRPD data.

### Solubility Studies

In this study, the solubility of the drug alone in different pH values simulating pH of GIT was studied: pH 1.2 (gastric pH), pH 6.5 (small intestine pH) and pH 7.2 (colon pH). The results summarised in Table 3 indicate that the solubility of gefitinib is pH dependent. The drug is relatively highly soluble in the gastric pH, compared to intestinal and colonic pH values.

**Table 3:** Solubility of ZD at Different pHs. Mean ± SD, ***n***=3

pH	Solubility (μg/mL)	Log solubility
1.2	1000±1.6	3.0±0.2
6.5	11±1.25	1.04±0.1
7.2	10±1.12	1.0±0.05

Table 3 shows the solubility of ZD (μg/mL) at pH values 7.2, 6.5 and 1.2. Solubility data are provided in both linear and logarithmic forms.

### Dissolution Studies

**Fig 5:**
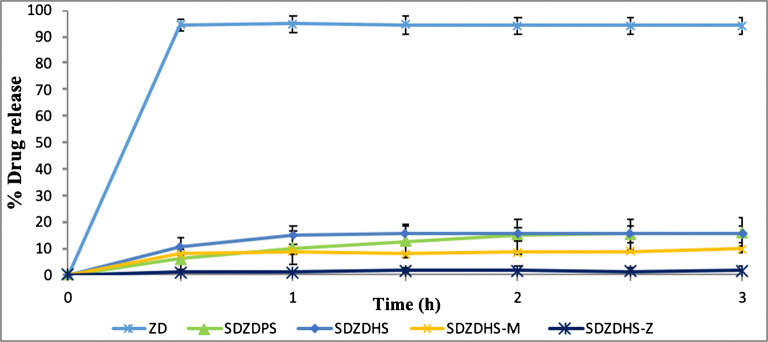
The dissolution profile for Crystalline Gefitinib and spray-dried formulations into pH 1.2; mean ± SD, three independent batches were analysed, each 6 times (***n***=18)

Figure 5 shows dissolution profiles of pure and solid dispersion formulations at pH 1.2. ZD used as a reference in dissolution studies is a weak base with two pKa values of 5.4 and 7.2. At low pH, ZD is fully ionised and its water solubility is high. Nearly all (94%) of pure ZD dissolved within half an hour at pH 1.2. On the other hand, SDZDPS and SDZDHS solid dispersions released only small percentages of the loaded drug, typically 16% and 15.5% respectively at pH 1.2, over a relatively longer time period of 3 h (Figure 5). These behaviours could be ascribed to effect of polymeric matrices of PVP, HPMC and Eudragit S 100 on the release of ZD. In order to achieve targeted colon delivery, a further suppression of drug release at pH 1.2 was needed. Therefore, the SDZDHS solid dispersion was optimised by increasing the Eudragit S 100 (hydrophobic polymer) content (see Table 1 for quantitative composition) to further suppress drug release at pH 1.2. The optimised formulation (SDZDHS-M) showed an enhanced retardation of drug release at pH 1.2 as compared to SDZDHS. Increasing the Eudragit S 100 content (i.e. formulation SDZDHS-Z) would potentially promote better colonic targeting and performance.

**Fig 6.**
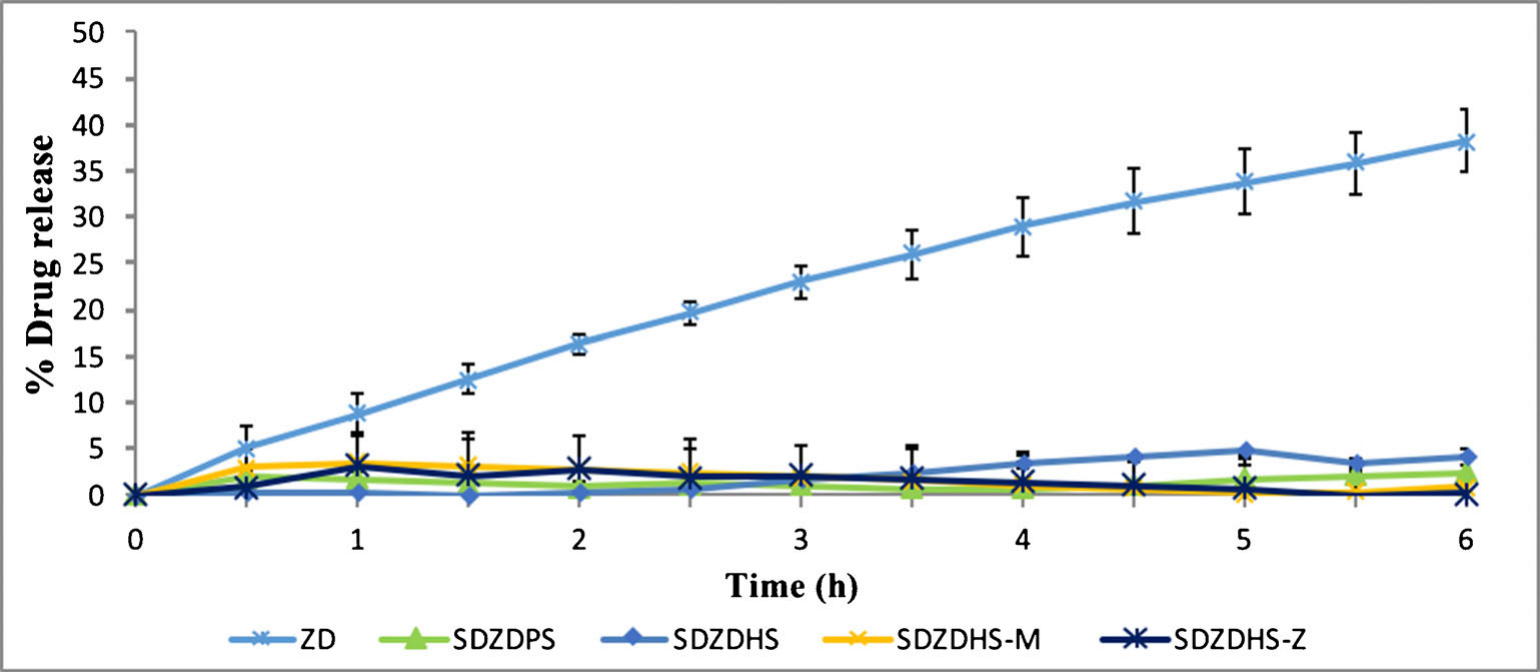
The dissolution profile for Crystalline Gefitinib and spray-dried formulations into pH 6.5; mean ± SD, three independent batches were analysed, each 6 times (***n***=18)

Figure 6 shows dissolution profiles of pure and processed ZD at pH 6.5. Only 38.3% of pure drug was released in pH 6.5 after 6 h. Lower dissolution extent and rate were recorded for the pure drug at pH 6.8 compared to that at pH 1.2. This pH-dependent behaviour is due to lower percentage of ionisation and hence lower solubility and dissolution of this basic drug (ZD) at the higher pH values, compared to acidic pH where the drug is almost completely ionised. Further retardation in dissolution rates was recorded for SDZDPS, SDZDHS, SDZDHS-M and SDZDHS-Z solid dispersion. Only 2.5%, 4.1%, 0.9% and 0.2% of loaded drug were released at pH 6.5 (after 6 h), respectively. The presence of Eudragit S100 plays a crucial role in potential colon targeting by preventing the drug release at segments of the GIT other than the colon.

**Fig 7:**
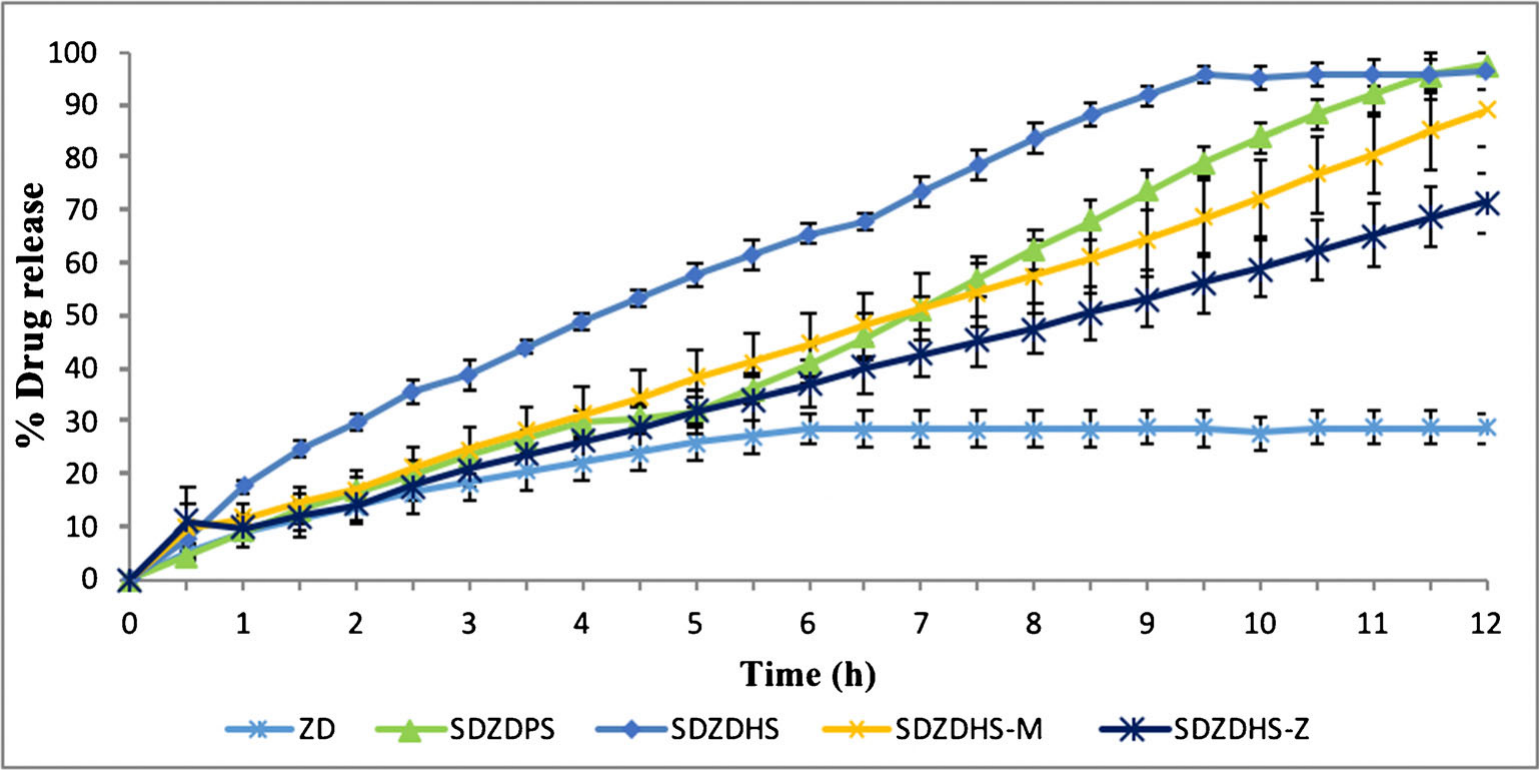
The dissolution profile for Crystalline Gefitinib and spray-dried formulations into pH 7.2; mean ± SD, three independent batches were analysed, each 6 times (***n***=18)

Figure 7 shows the dissolution profiles of pure and processed ZD at pH 7.2. Only 28.7% of pure drug was released in less than 12 h in pH 7.2. SDZDPS, SDZDHS and SDZDHS-M solid dispersion released 96.7%, 76.7% and 89% of loaded drug at pH 7.2 (after 12 h), respectively. Increasing the ratio of Eudragit S (i.e. formulation SDZDHS-Z) displayed the best colonic controlled-release performance as shown in (Figures 5 and 6). However, the increase in Eudragit S 100 content resulted in a slower release rate at pH 7.2 as shown in (Figure 7); therefore, the run time was extended for this formulation to 15 h to allow a maximum drug release to take place (data not shown). Increasing Eudragit S100 concentration is likely to produce a denser polymeric matrix in order to increase the production at low pH, and hence, more time is required for polymer to dissolve and release drug molecules.

Almost 100% of the drug was released from the SDZDHS-Z solid dispersion within 15 h at pH 7.2 (data not shown). HPMC 603 and PVP were used to enhance the drug dissolution in the first place, while the role of Eud S 100 was to prevent the drug release in the upper GIT. The solubility of the drug at basic pH seems to be very low and this partly contributes to prolonged dissolution rates. Similar results were reported with HPMC colon targeted paracetamol capsules with two enteric polymers Eudragit L 30 D-55 and Eudragit FS 30 D. The dissolution studies showed gastric resistant release for 2 h at pH 1.2 and capsules coated with Eudragit FS 30 D were resistant for a more 1 h at pH 6.8 (35).

### Mucoadhesion Study

PVP is one of the most commonly used polymers to make solid dispersions, hence chosen to enhance the dissolution of the poorly water-soluble drug; ZD (36). However, there is no evidence in the literature to suggest that PVP is mucoadhesive. Mucoadhesion happens when a substrate is attracted (via various forces) to a mucus layer that coats the epithelium of a tissue. Mucoadhesiveness is a useful characteristic as it can potentially prolong retention time of a formulation at a target site and maximise the contact time with the bio-absorption site to increase drug permeability through biological membranes(21). In this study, the water-soluble carrier (HPMC) was employed to enhance the mucoadhesion properties of the optimised formulation. This is likely due to availability of hydroxyl groups (OH) groups to form H-bonding and electrostatic attractions with mucin. The rotating cylinder method was used to estimate the time of adhesion to the tissue of formulated PVP, and HPMC solid dispersions in the presence of Eudragit S100 (24). The evaluation of adhesion of the formulations depends on the time that the glass slide remains in contact with the tissue. The longer the adhesion time, the stronger the mucoadhesion forces.

Table 4 provides a summary of mucoadhesion time of all tested formulations at the pH values of interest. While there was no significant difference in the retention times (*P*-value = 0.09 and 0.37 respectively) between SDZDPS and SDZDHS formulations at pH 1.2 and pH 6.5 (relatively poor adhesion at gastric and upper GIT pH), a significant increase of retention times (*P*-value=0.00016) was observed at the pH 7.2 (Table 4). Indeed, Eudragit S100 seems to have a protective effect at lower pH where it prevents the system from dissolving, consequently limiting mucoadhesion. Further, at higher pH > 7, the carboxylate groups of mucin are fully ionised and can better interact with HPMC. In addition, as the pH increases above the dissolution threshold for Eudragit S (> pH 7), SDZDHS (containing Eudragit and HPMC) showed a higher duration of adhesion (1188 min) comparing with PVP containing SDZDPS (269 min). This synergistic effect can be possibly attributed to the ability of HPMC to swell on contact with aqueous media, causing increases in the unfolding of the polymeric network and free chain mobility resulting in an increase in polymer-mucin interactions by entanglement and/or by hydrogen bonding (21). Such pH-dependent mucoadhesion profile is expected to specifically prolong retention time of ZD at the target site. This is important to maximise the retention of ZD formulation at the ascending colon where fluid is relatively more abundant allowing an enhanced dissolution and absorption. Therefore, HPMC was used instead of PVP in solid dispersion formulations with ZD and Eudragit S 100 to carry out further optimisation. The longer the adhesion time, the better retention where more time is allowed for drug uptake by target tissues. For example, xanthan gums, chitosan and polyethylene oxide polymers recorded adhesion times of 153.5, 43 and 89 min respectively (37). However, the adhesion time recorded for SDZDHS was approx. 20 h. This is a relatively lengthy time for an *ex vivo* study that might lead to some changes in properties of the excised tissue, a matter that needs to be taken into consideration when interpreting these results. Nevertheless, these findings indicate that HPMC has superior mucoadhesive properties compared to PVP. PVP is one of the most commonly used polymers with solid dispersions, hence chosen to enhance the dissolution of the poorly water-soluble drug; ZD (36). Interestingly, there is no evidence in the literature to suggest that PVP is mucoadhesive.

**Table 4:** Duration of Mucoadhesion of Solid Dispersion Formulations at Different pHs

Formulation		Adhesion time (min)	
	pH 1.2	pH 6.5	pH 7.2
SDZDPS*	7±1.0	5±0.6	269±41.8
SDZDHS*	9±1.5	6±1.0	1188±153.7

*P, PVP; *H*, HPMC; *S*, Eudragit S 100

### Cell Viability Assay

**Fig. 8:**
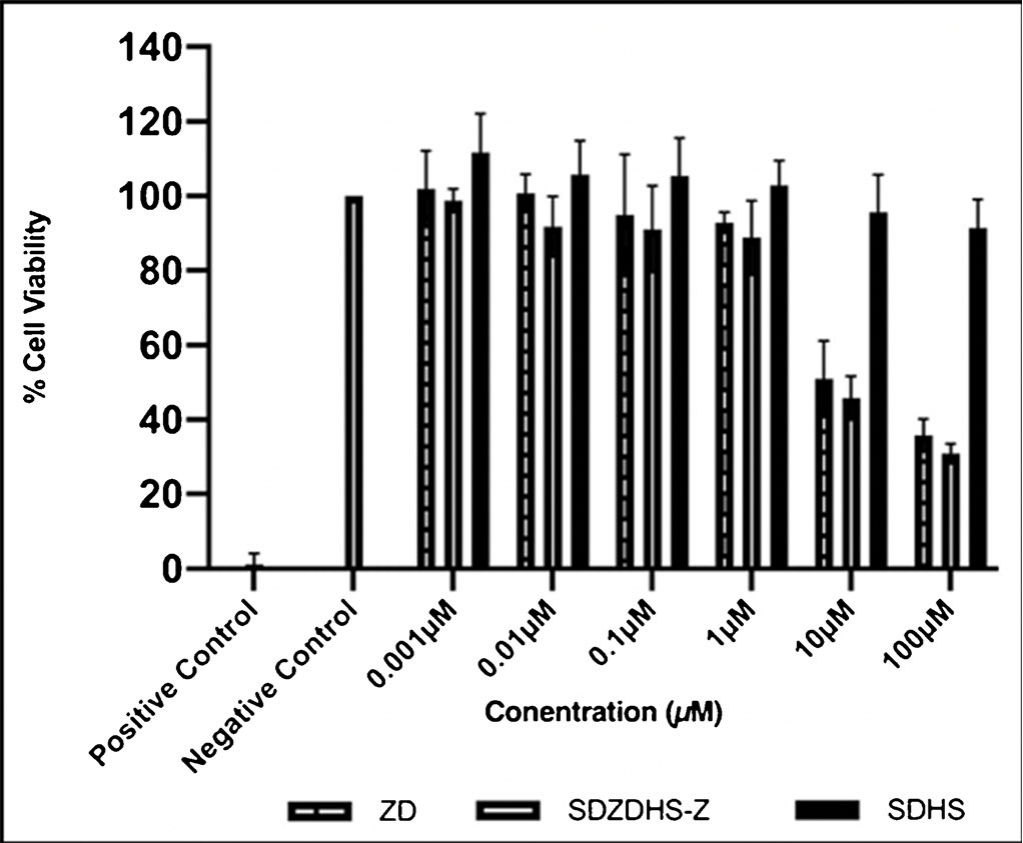
The effect of ZD, SDZDHS-Z and SDHS on the growth of Caco-2 cell after 24 h using the neutral red; ***n***=3

Neutral red uptake is one of the most commonly used viability assays that provide a quantitative estimation of the number of viable cells. This test is reliable and provides fast background absorbance when measured in the absence of cells. Furthermore, it has been reported to be more sensitive and cheaper than other tetrazolium salts-based cytotoxicity assays (38). Viable cells have the ability to incorporate and bind the neutral red supravital dye in lysosomes, which is later extracted (38). This test has been successfully employed to evaluate the cytotoxicity of polyelectrolyte nanocomplex of chitosan and hyalurnoic acid for colon delivery of insulin (39). In this study, caco-2 cell lines were dosed, and the treatment range was picked up in accordance with the IC_50_ value of 0.033 μM for pure ZD under the conditions and exposure times as previously described (40). Serial dilutions of drug-free polymeric dispersions (SDHS), drug (ZD) and drug-loaded spray-dried dispersions (SDZHS-Z) of different concentrations (0.001 μM, 0.01 μM, 0.1 μM, 1.0 μM, 10 μM and 100 μM) were tested for cell viability against positive and negative control (Figure 8). Cell viability (%) recorded for SDHS (drug free polymeric dispersions) at concentrations from 0.001 to 100 μM was 100% indicating high tolerability and no cytotoxicity to the excipients used. On the contrary, concentration-dependent cytotoxicity was reported to drug alone and drug formulated as spray-dried dispersion. These results indicate that the used excipients/polymers used had no cytotoxic effects comparable to the negative control and hence can be considered as non-toxic (41). However, it is worthwhile noting that the HPMC solid dispersion loaded with drug (SDZDHS-Z) are likely to remain in contact with the target tissue (colon) for prolonged time period (as demonstrated by the mucoadhesion results (previous section)), which along with the improved dissolution and extended release of ZD could potentially translate to improved efficacy *in vivo*. Hence, the importance of further proof-of-concept studies to be conducted *in vivo* using an appropriate animal model.

Figure 8 shows the percentage of cell variability (Caco-2) with increasing concentrations of pure ZD, HPMC solid dispersion loaded with drug (SDZDHS-Z) and blank formulation (SDHS). It is apparent that treatment with pure drug and formulated drug as solid dispersion causes Caco-2 cell growth inhibition. The response of Caco-2 cells to treatment appears to be dose-dependent with comparable growth inhibition results for both pure (ZD) and processed drug (SDZDHS-Z) at all concentrations (*P* > 0.05). This indicates that the used excipients and the formed solid dispersions do not interfere with cytotoxic effects of the drug. Further, it is worthwhile mentioning that while the formulation tested (SDZDHS-Z) was not superior to the pure drug (ZD) in inhibiting cell growth, one needs to remember that cell culture studies are conducted in an environment that would not allow the desirable mucoadhesive properties of (SDZDHS-Z) to affect the formulation’s performance. Hence, the need to further investigate these formulations in an appropriate *in vivo*(animal) model.

## CONCLUSION

In this study, we reported (for the first time) on polymeric formulations of ZD that were prepared by a simple and scalable spray drying method. Formulations based on HPMC and Eudragit S 100 showed an enhancement in dissolution and mucoadhesion of (ZD) which could potentially result in improved colonic delivery of ZD. The spray drying method was successfully employed to preparer amorphous micronised spherical particles of ZD in polymeric carriers comprising PVP or HPMC with Eudragit S 100. The pH-dependent dissolution profiles and mucoadhesion characteristics were demonstrated by all formulations with the HPMC-based solid dispersion being the most promising. The neutral red assay results conducted on a Caco2 cell line showed a dose-dependent response which was not affected by the polymers or spray drying process used. Yet, the HPMC-based drug-free formulation had no toxic effects on cells. This can warrant further investigation of the prepared formulations in an appropriate animal model where the effect of mucoadhesion on efficacy could be established.

## Abbreviations

Eud: Eudragit S100
HPMC: Hydroxypropyl methyl cellulose
PVP: Polyvinylpyrrolidone
SD: Spray dried
ZD: Gefitinib
